# Collision Every Night: Treating Nightmares with Trauma-focused Methods: Case Report

**DOI:** 10.1007/s41782-021-00126-8

**Published:** 2021-02-01

**Authors:** József Szabó, Szilvia Tóth

**Affiliations:** 1 Faculty of Humanities and Social Sciences, Pázmány Péter Catholic University, Mikszáth Kálmán tér 1, Budapest, 1088 Hungary; 2 Department of Psychiatry, Insomnia Outpatient Service, St. Raphael’s Hospital in Zala County, Zrínyi M. u. 1, Zalaegerszeg, 8900 Hungary; 3 Department of Psychiatry, St. Raphael’s Hospital in Zala County, Zrínyi M. u. 1, Zalaegerszeg, 8900 Hungary

**Keywords:** Psychotrauma, Acute stress disorder, Nightmare, EMDR, Imagery rescripting

## Abstract

**Introduction:**

We would like to present the case of a young patient with acute stress disorder and recurrent nightmares following the psychological trauma caused by a severe road traffic accident. The comprehensive therapy carried out at the Department of Traumatology included medication, trauma processing and a psychological method whose aim is to cease the development of nightmares.

**Case Presentation:**

Psychiatric assessment and treatment was asked for a polytraumatised female patient at the Intensive Care Unit after she had undergone a neurosurgical intervention. Her medicinal treatment was continued at the Department of Traumatology. Besides the antidepressant venlafaxine she was treated in accordance with the EMDR protocol for acute stress disorder, and we also applied imagery rescripting to prevent her from having recurrent (daily) nightmares. As a result of the therapy, her symptoms were fast relieved, the nightmares stopped almost instantly, her mood improved, rumination and anxiety decreased significantly.

**Conclusions:**

In view of the fast and significant symptomatic improvement, we can expect that the EMDR therapy and its protocol for acute stress disorder have successfully reactivated information processing, and besides the subjective relief we have managed to prevent a mental crisis that could lead to a suicide risk as well as the development of post-traumatic stress disorder. We also hope that the improvement will be long-lasting.

## Introduction

Our case presentation summarises the consultation-liaison psychiatric management of A. A., a 31-year-old female patient. Our paper has a twofold aim: to draw attention to the importance of this branch of psychiatry [1] through the introduction of a successful therapy and also to give an example of the practical application of trauma-focused psychological approaches, which are developing dynamically. Our patient sustained serious injuries in a road traffic accident that she caused herself, and one of the most dramatic circumstances of the accident was that her children (passengers in the car) also got injured. Her younger child [2] suffered minor injuries while the older one [3] got seriously injured. At the time of our first meeting, the child could not walk or speak and was cared for at the Department of Paediatrics. Both the local and the national media reported on the accident. Immediate trauma-focused therapy was considered essential to provide immediate relief for her subjective suffering and also to prevent the development of psychotrauma-related disorders (PTSD, reactive depression, etc.) in the long run.

## Background

According to scientific literature, 21% of the survivors of road traffic accidents experience acute stress reaction. At the scene of disasters or accidents, the members of the rescue team can usually detect acute stress in 10% of the survivors and eyewitnesses. The same proportion of people develop posttraumatic stress disorder (PTSD) a few months later, and there is some overlap of the two groups suggesting some kind of connection; however, this relationship has not been proved yet. Some studies have concluded that the two phenomena are independent of each other, while others consider untreated acute stress disorder as the main cause of PTSD [4]. Some authors argue that their research has found a link between acute stress disorder and completed suicide. In their opinion, those suffering from depression or chemical dependence often end their life as a result of acute stress disorder [2]. Acute stress and PTSD developing after life events that qualify as psychotrauma are often accompanied by sleep disorders, most commonly insomnia and nightmares [5].

When treating our patient, we tried to complete the medicinal treatment with a therapy that has been proved effective in the treatment of mental disorders caused by psychotrauma. One such approach is the trauma-focused, integrative, almost eclectic psychotherapy called Eye Movement Desensitization and Reprocessing (EMDR) whose primary aim is the processing of traumatic memories that underlie several mental disorders. As for the technical aspect, its central element is horizontal eye movement (or some other alternate side stimulation). After recalling traumatic memories and the accompanying emotional state, patients can develop a relaxed state of mind as a result of rhythmic eye movements. This relaxed state of mind reduces the negative emotional charge of the traumatic memories and creates long-lasting behavioural changes**.** Emotions associated with the memory will be less intense, subjective suffering will decrease and the traumatic memories will resurface less frequently [6].

EMDR psychotherapy was first introduced in 1989 but it was only much later when it became recognised and widely used. In Hungary it debuted only after 2000. In the meantime, its effectivity has been confirmed by several clinical trials. Nowadays it is becoming more widespread used in several psychological traumas other than PTSD, and its application is facilitated by disorder-specific treatment protocols [7].

We chose the *acute stress protocol* of EMDR to treat acute stress reaction or acute stress disorder. Nowadays it is a widely used approach in the treatment of PTSD and other mental disorders accompanying psychological traumas [8]. One part of our intervention was carried out along the EMDR protocol for acute stress disorder [4, 9].

The other method we applied was *imagery rehearsal therapy*, which a particular type of imagery rescripting frequently used in the treatment of nightmares. Imagery rescripting [3, 10, 11] is well established in the treatment of several mental disorders, primarily anxiety disorders, either alone but most frequently supplementing other techniques (e.g., schema therapy). Imagery rehearsal therapy, which addresses nightmares frequently developing as a characteristic feature of mental disorders following psychotrauma, is an excellent supplement to other trauma-processing methods [12, 13]. As part of the therapy, we ask the patient to memorise or even record their dream. During the session, they are asked to change the story so that it has another, more acceptable and tolerable, less negative ending. They are supposed to imagine the details of the rewritten dream every night before falling asleep or immediately after waking up if the nightmare recurs. It will help going back to sleep after waking up, and the nightmares will gradually cease. The approach is also part of comprehensive sleep therapy as it is effective not only in PTSD but also in idiopathic nightmares [14, 15]. Nightmares, especially if they become chronic, are notorious for significantly impairing the quality and quantity of sleep. People with chronic nightmares often show daytime symptoms of lack of sleep typical of insomnia, which significantly impair both mental and physical health in the long term [13].

## Case Presentation

The patient’s psychiatric assessment for depressive symptoms was first requested by the Intensive Care Unit at our hospital. According to the letter of referral, 2 weeks earlier she had suffered a road traffic accident and became polytraumatised as the driver of a passenger car The patient received neurosurgical care in another county, then was transferred to our hospital,’ where her level of consciousness was gradually improving and subsequently she developed depressive symptoms’. The Intensive Care Unit asked for therapeutic recommendations.

The psychiatric assessment was carried out on 17 April, 2020. The past medical history of the patient was not significant for any psychiatric disorder. During the psychiatric interview, she stated that she had found it increasingly difficult to deal with not being able to recall what had happened to her and not knowing whether the accident had been her fault. She was constantly ruminating about the accident and could not think about anything else. She was also concerned about not being able to see her children as they had never been separated earlier: she was still on maternity leave since the birth of her son. She was having nightmares but she could not recall them; she always woke up crying and disturbed by negative feelings. She did not use to cry earlier, but on the day of the interview she was very upset. She had no suicidal thoughts but she was impatient, eager to recover and return home to her family. She felt pleased about being able to move as lying down and immobilisation had also been stressful for her. She was looking forward to her potential transfer to the Department of Traumatology.

### Mental state

Alert and oriented in all spheres. Her attention shows excessive tenocity but it can be raised and diverted. Her way of thinking can be characterised by limited associations, guilt and rumination. No delusions or perceptual disorders can be detected. Her cognitive function is intact; however, she cannot recall the accident and what happened immediately afterwards (lacunar amnesia). Her mood is generally low with unstable emotions. She reports sleep maintenance insomnia, dyssomnia, as well as increased anxiety. She is cooperative, not suicidal and has appropriate insight.

### Diagnosis

F3290. Unspecified depressive episode. Recommendations: one venlafaxine 50 mg o.m., individual trauma-focused Cognitive Behavioural Therapy after the patient is transferred to the Department of Traumatology.

We could not use such instruments, as usual (for example Beck Depression Inventory, Beck Anxiety Inventory) in our differential diagnostic activity, because of her injuries, and the circumstances of intensive care. So our diagnosis was based on only the criteria of DSM-5.

The first session of the individual, trauma-focused therapy was held in a single-bed ward of the Department of Traumatology a few days later. At that time, the patient was still not able to get out of the bed and even changing her body position caused significant pain. Intensive physiotherapy was initiated as part of the rehabilitation. During the session, the patient revealed that she had no recollection of the accident, the details of which had been described to her by her husband, who was not involved in the collision. She reported extreme sadness and helplessness, furthermore she was not able to speak about the accident, the actual situation, their children’s condition or the near future with her husband, consequently his visits were characterised by long silence. Her days are spent ruminating about the future, the accident and the events preceding the accident. At night she is tortured by nightmares, crying, screaming, tossing violently but not waking up. Usually it is the nurses working on the night shift who have to wake her up since her intensive movements could have a negative impact on her physical injuries. She always reports the same dream: she is sitting in her car, behind the steering wheel, the car is stationary and the children are crying on the back seat. The same thing happens every night. After waking up, she is scared and usually cannot go back to sleep for approx. an hour. During the day, she often feels exhausted but tries deliberately to stay awake and maintain the normal sleep-wakefulness cycle hoping for a relaxing sleep. Anyway, both her daytime and nighttime sleep are significantly hindered by worrying and rumination. The symptoms detected and reported at the time of the session met the criteria of acute stress disorder.

A blessing in disguise was that both the patient and her husband are employed by our hospital, so in spite of the COVID-19 emergency restrictions and the ban on inpatient visits they could keep in touch with each other. Her husband was understanding and supportive not blaming her for the accident.

In the hope of immediate relief evoked by the eye movement desensitization of EMDR, the processing of the traumatic event was carried out during the very first session. As the patient could not recall the accident itself, we chose the moment when she first learnt about the accident, her injuries and her children’s condition. The patient was given an approx. 10-min psychoeducation about stress, psychological trauma, EMDR therapy and its information processing model. In accordance with the acute stress therapy protocol (R-TEP) of EMDR, the patient was asked to retell the traumatic events while we performed continuous bilateral simulation (in this case it was alternate tapping of the hands while the patient was lying in her hospital bed) with the aim of integrating the traumatic memory fragments into a narrative [9]. The patient was also asked to mentally relive the events as much as possible. In accordance with our expectations, the emotional and physical components of the stress reaction experienced during the trauma reoccurred as part of the recollection, and their intensity almost reached that of the first trauma.

In the next step, we used the so-called Google-Search procedure (Elan Shapiro) to identify the most critical moment of the traumatic event, when the patient experienced the most intensive emotional stress. During the procedure, the patients are undergoing continuous bilateral stimulation and at the same time they are asked to mentally replay the traumatic event and stop the memory at the most critical moment. We will use the feelings and physical sensations of this inner representation of the traumatic event for the eye movement desensitisation of the EMDR therapy [9]. As the moment identified this way is either extremely painful or it is the most painful moment of the whole memory, and since neither the past history of the patient nor the targeted questions of the EMDR therapy suggested further unprocessed psychotrauma, we considered the accident as the primary trauma (not trauma repetition). Of the well-established three options, we chose the simplest and fastest technique, EMD. If the case had involved trauma repetition (and posttraumatic disorder accompanying the acute stress reaction), we should have performed the more comprehensive, full EMDR protocol, and if the traumatic event had included more than one critical moment, the EMDr strategy should have been chosen [9]. In the latter case, each critical moment has to be recalled and desensitised separately. In this case, the most critical moment (Point of Disturbance, PoD) was the time when the patient woke up in the Intensive Care Unit and realised her own condition then learnt the details of the accident from her husband’s report. The patient experienced a wide range of emotions whose intensity was very similar to that of the original ones: fear, desperation, helplessness, anger, sadness, disappointment, remorse. She described her physical sensations as chest, stomach and throat tightness, as well as shortness of breath.

First we chose the classic eye movement for desensitisation; however, the patient’s eyes became tired and painful very fast. As a result, we switched to alternate side tapping, which was performed on the hands of the patient while she was lying in the hospital bed, her hands on the duvet. The emotional and physical constituents of the elicited stress reaction could be reduced to minimal relatively quickly; after only two cycles of bilateral stimulation [9]. As a result of desensitisation (bilateral stimulation), the initial intensity of emotional and physical distress rapidly decreased to zero from 10 (measured with the Subjective Unit of Disturbance Scale, SUDS, a scale first used in behavioural therapy but nowadays frequently applied in EMDR as well) [16, 17]. When the critical moment was recalled, the score did not increase above 1 either. She used to word *miserable* as the adjective to best describe herself, which included both meanings of the word: unhappy/unfortunate as well as of low value/quality. According to her, she did not use to be like that; even though being a bit hesitant, she had always been able to control her life, make appropriate decisions, make plans and realise them. In the end, it was the control over her life, as well as her knowledge and experience of this control that we strengthened with slow eye movement in the Installation phase [8, 18].

Some degree of muscle tension developed in the next, Body Scan phase [6] but it also responded to bilateral stimulation and the patient became relaxed, reporting relief and calmness. Recalling the traumatic event did not evoke the previous intense emotional response; she only reported slight sadness but felt relaxed. Apart from being relieved, she also felt sleepy and was looking forward to a relaxing sleep.

During the second session 4 days later she said that the relief she had experienced after the first sensitisation session turned out to be long-lasting and she was now able to communicate with her husband. Earlier she had found it very difficult but now they were talking about the condition of their children, their plans and the future. This regained ability further increased her relief. However, she was still troubled by the nightmares, thus we started to acquire the imagery rescripting technique to focus on the dreams. Before addressing the actual content of the dreams and trying to change them, we performed the *safe place* protocol, which is a technique combining relaxation, imagination and meditation. This tool can create a feeling of safety, and following acquisition patients can perform it on their own to decrease anxiety and restore the feeling of safety. This technique can be a vital part of every integrative therapy aiming to treat psychological traumas [19]. The calmness and relative safety thus created provided a suitable atmosphere for the next step. The imaginary safe place in this case was connected to a real experience and a real place. It was a place the patient, her husband and their friends had visited on holiday before their children were born. She had long been planning to return there once with the children. Then we recalled the recurrent nightmare described above and the applied imagery technique created a very sharp image and intense feelings. The patient’s throat tightened and her eyes started tearing. We had the following conversation:

Therapist: Where are we? What’s going on?

Patient: We’re sitting in the car, the three of us. The kids and me. They’re crying in the back seat.

P: It must be after the crash…

T: Cannot it be anything else?

P: Well, my kids are not the crying type.

T: Have they never cried in the back seat?

P: Rarely.

T: What made them cry?

P: They were cross.

T: Why?

P: They were hungry, thirsty or needed to pee.

T: Cannot that be the problem now as well?

P: Maybe.

T: What shall we do then?

P: We always have some snacks and drinks with us. I’ll offer them some, then we’ll get out, walk and pee. Get some air.

T: Then what?

P: We’ll get back in the car and go on.

T: Where are you going?

P: I don’t know.

T: What is the weather like?

P: Nice, sunny and warm?

T: Maybe you’re going on holiday?

P: But my husband is not here.

T: What can that mean?

P: Maybe he’s on duty and will join us later.

T: We’re getting closer. This is the place where you were on holiday with your husband earlier.

P: Yeah, I can see it now. And there is my husband, waiting for us.

T: What’s the place like?

P: Pleasant, just like it was ten years ago when it was just the two of us.

T: How are you feeling now?

P: Good, I’m relieved.

At that time, I asked the patient to replay this new, imaginary dream every night when lying in the bed relaxed (she had learnt relaxation techniques earlier). Should she have another nightmare, she should replay it once more after waking (or being woken) up to ease the scare, then try and get back to sleep. This took place on a Friday.

## Results

Our next session was scheduled for next Tuesday. The patient reported that in spite of being rather sceptical about the benefits of the method, she had been working hard and replayed the new dream several times a day, not only prior to going to sleep. Although she had not trusted the nightmares to stop, she was surprised to find that they had not bothered her during the first night, nor has she had any since then. We agreed that she would continue with the methods for a few weeks and resume replaying the dream should the nightmares ever present in the future. As the memory processing performed along the EMDR protocol for acute stress disorder caused her anxiety, worries, rumination and helplessness to decrease significantly and her mood had improved as well, there was no need for another stress-relieving intervention. Her other difficulties arising from her special situation at the time were addressed from a cognitive viewpoint during the session. During the next session on the next week, she did not complain of any severe symptoms, therefore the therapy was summarised and ended. Naturally, the therapy can be resumed if her condition changes. Another week later we paid her a short visit and found that her physical condition had been improving as well.

Not long after her attending physical called to inquire about withdrawing her antidepressant in view of the significant symptomatic improvement. She received an oral recommendation concerning the duration of the medicinal therapy and the dosage of the medicine.

## Conclusions

We have to mention as a limitation, that we could not use psychological inventories in our differential diagnostic work. Although the fast and significant improvement in the patient’s condition suggests complete processing of the psychological trauma, we cannot be absolutely certain since stress-related disorders (psychosomatic diseases, depression, PTSD) do not tend to develop immediately after the trauma. As a result of the short time, we cannot be certain and definitely do not wish to predict the further course of the patient’s psychological trauma. However, we do hope that the EMDR therapy and its protocol for acute stress disorder have successfully reactivated information processing and besides the subjective relief we have managed to prevent a mental crisis that could lead to the above mentioned psychological disorders and the risk of suicide. Taking the elapsed time into account we are confident enough to state that we have succeeded in preventing a mental crisis. We have definitely prevented the nightmares from becoming chronic and could also avoid its potential psychological and somatic consequences.
